# New insights into the domain of unknown function (DUF) of EccC_5_, the pivotal ATPase providing the secretion driving force to the ESX-5 secretion system

**DOI:** 10.1107/S2059798324004248

**Published:** 2024-05-28

**Authors:** Fernando Ceballos-Zúñiga, Margarita Menéndez, Inmaculada Pérez-Dorado

**Affiliations:** a Department of Crystallography and Structural Biology, Institute of Physical Chemistry Blas Cabrera, Spanish Research Council, Serrano 119, 28006 Madrid, Spain; b Department of Structure and Thermodynamics of Macromolecules, Institute of Physical Chemistry Blas Cabrera, Spanish Research Council, Serrano 119, 28006 Madrid, Spain; c CIBER of Respiratory Diseases, ISCIII, Sinesio Delgado 10, 28029 Madrid, Spain; University of Queensland, Australia

**Keywords:** ESX systems, DUF domain of EccC_5_, T7SS, secretion systems, mycobacteria, *Mycobacterium tuberculosis*, ATPase activity, crystallography

## Abstract

Type VII secretion (T7S) systems, also referred to as ESAT-6 secretion (ESX) systems, are molecular machines that have gained great attention due to their implications in cell homeostasis and in host–pathogen interactions in mycobacteria. The latter include important human pathogens such as *Mycobacterium tuberculosis* (*Mtb*), the etiological cause of human tuberculosis, which constitutes a pandemic accounting for more than one million deaths every year. The ESX-5 system is exclusively found in slow-growing pathogenic mycobacteria, where it mediates the secretion of a large family of virulence factors: the PE and PPE proteins. The secretion driving force is provided by EccC_5_, a multidomain ATPase that operates using four globular cytosolic domains: an N-terminal domain of unknown function (EccC_5_^DUF^) and three FtsK/SpoIIIE ATPase domains. Recent structural and functional studies of ESX-3 and ESX-5 systems have revealed EccC^DUF^ to be an ATPase-like fold domain with potential ATPase activity, the functionality of which is essential for secretion. Here, the crystal structure of the *Mtb*EccC_5_^DUF^ domain is reported at 2.05 Å resolution, which reveals a nucleotide-free structure with degenerated *cis*-acting and *trans*-acting elements involved in ATP binding and hydrolysis. This crystallographic study, together with a biophysical assessment of the interaction of *Mtb*EccC_5_^DUF^ with ATP/Mg^2+^, supports the absence of ATPase activity proposed for this domain. It is shown that this degeneration is also present in DUF domains from other ESX and ESX-like systems, which are likely to exhibit poor or null ATPase activity. Moreover, based on an *in silico* model of the N-terminal region of *Mtb*EccC_5_^DUF^, it is hypothesized that *Mtb*EccC_5_^DUF^ is a degenerated ATPase domain that may have retained the ability to hexamerize. These observations draw attention to DUF domains as structural elements with potential implications in the opening and closure of the membrane pore during the secretion process via their involvement in inter-protomer interactions.

## Abstract 2

The crystal structure of the DUF domain of EccC_5_ from *Mycobacterium tuberculosis*, a degenerated ATPase domain with potential implications in the opening and closure of the membrane pore in the *M. tuberculosis* ESX-5 secretion system, is reported.

## 1. Introduction

Molecular machines are sophisticated protein complexes that are ubiquitously present in all cellular organisms (Miller & Enemark, 2016 ▸). They convert the chemical energy resulting from the hydrolysis of nucleoside triphosphates (NTPs) into the mechanical force needed in numerous cellular events such as DNA replication, protein degradation, cell motility and protein secretion, amongst others (Miller & Enemark, 2016 ▸; Schmidt *et al.*, 2012 ▸; Famelis *et al.*, 2023 ▸; Crosskey *et al.*, 2020 ▸). The members of the superfamily of ATPases associated with diverse cellular activities (AAA+ proteins) are key constituents of many molecular machines, where the mechanical work is performed at the expense of adenosine triphosphate (ATP) hydrolysis (Miller & Enemark, 2016 ▸; Leipe *et al.*, 2002 ▸). Type VII secretion systems (T7SSs), also referred to as ESAT-6 secretion (ESX) systems, are AAA+-dependent molecular machines that are found in the *Actinomycetota* phylum and have gained great attention due to their implications in cell homeo­stasis and host–pathogen interactions in mycobacteria (Famelis *et al.*, 2023 ▸; Bunduc *et al.*, 2020 ▸; Houben *et al.*, 2014 ▸). The latter include devastating pathogenic species such as *Mycobacterium tuberculosis* (*Mtb*), the etiological cause of human tuberculosis (TB), which constitutes a pandemic that is responsible for more than one million deaths every year (World Health Organization, 2022 ▸). T7SSs are also found in bacteria belonging to the *Firmicutes* phylum (T7SSb), which include other important pathogens such as *Staphylococcus aureus*, *Listeria monocytogenes*, *Bacillus anthracis* and *B. subtilis* (Zoltner *et al.*, 2016 ▸; Mietrach *et al.*, 2020 ▸). Of note, T7Sb systems are distantly related to the T7Sa systems found in actinomycetes, thus constituting a divergent type of secretion systems.

**Figure 1:**
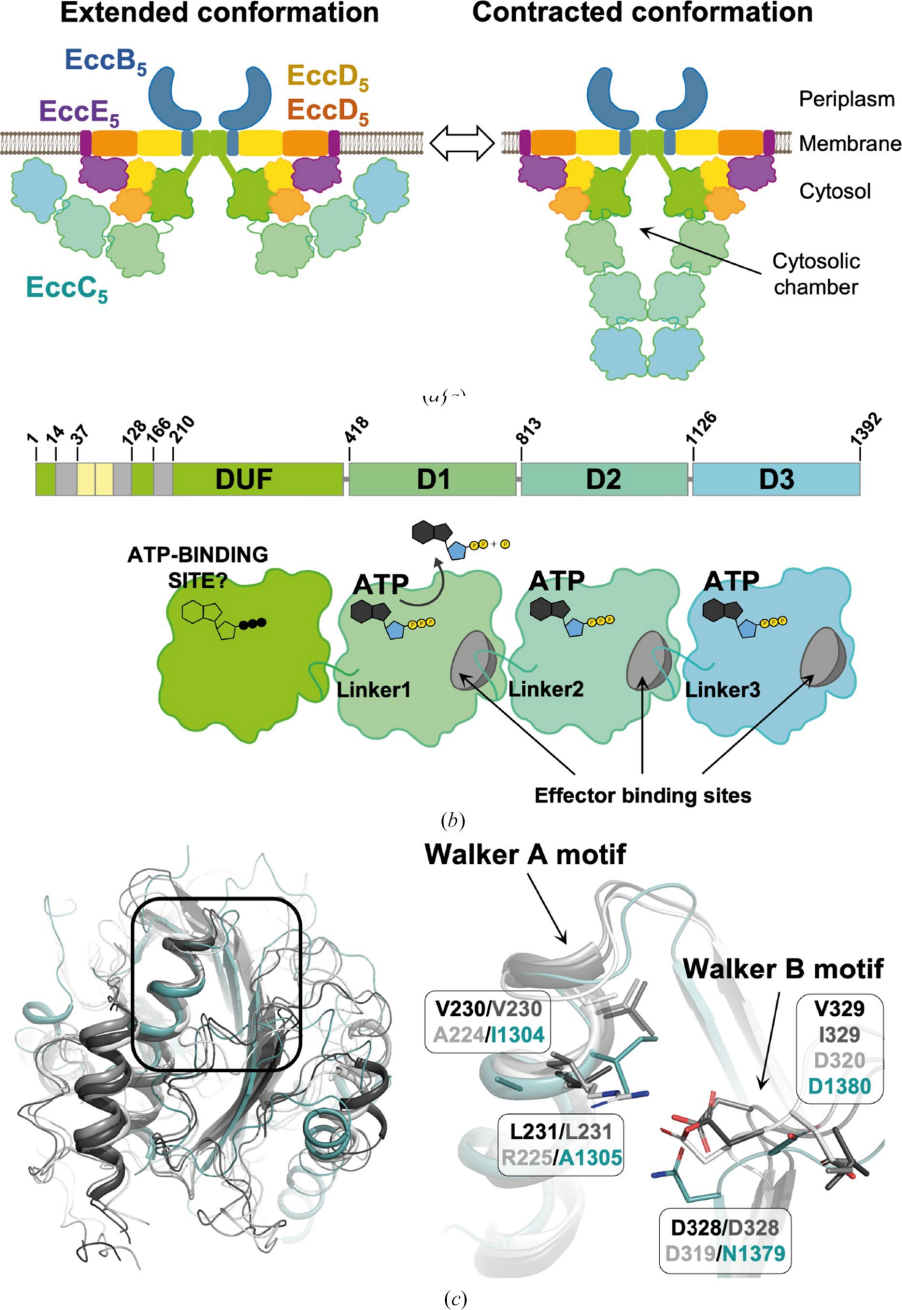
(*a*) *Mtb*ESX-5 model based on reported cryo-EM structures, showing the proposed conformational alteration of EccC_5_ between an open/extended conformation (left) and a close/contracted conformation (right). (*b*) Domain architecture of EccC_5_ consisting of the transmembrane (yellow), stalk (grey) and DUF (green) domains followed by three ATPase domains, D1, D2 and D3 (top), where the DUF and D1–D3 domains are interconnected by connectors referred to as Linker1, Linker2 and Linker3. (*c*) Superimposition of the *Srs*EssC^D3^ crystal structure (PDB entry 6vt1, blue) with cryo-EM models of 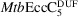 (PDB entry 7npr, dark grey), 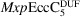 (PDB entry 7b9s, light grey) and 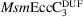 (PDB entry 6sgx, white) showing the conservation of the overall fold (left) and the similar arrangement of the Walker motifs (right).

Mycobacteria produce up to five ESX secretion systems (T7SSas), named ESX-1 to ESX-5, which consist of large membrane complexes that span the inner bacterial membrane. Among them, ESX-5 is found almost exclusively in slow-growing pathogenic mycobacteria, where it participates in nutrient uptake, intracellular colonization and modulation of the immune response during infection through the secretion of a large family of protein effectors: the PE and PPE proteins (Bunduc *et al.*, 2020 ▸; Houben *et al.*, 2014 ▸). These roles have important implications in the life cycle and virulence of the pathogens, thus indicating ESX-5 as a potential drug target (Bunduc *et al.*, 2021 ▸). Efforts aiming at structural–functional characterization of mycobacterial ESX secretion systems have resulted in structures of ESX-5 from *Mtb* and *M. xenopi* (*Mxp*) and of ESX-3 from *M. smegmatis* (*Msm*), which have illuminated the architecture of the pore complex (Famelis *et al.*, 2019 ▸; Poweleit *et al.*, 2019 ▸; Bunduc *et al.*, 2021 ▸; Beckham *et al.*, 2021 ▸; Fig. 1 ▸ *a*). The latter can be defined as a hexamer of protomers, in which each protomer is formed by four different membrane proteins, EccB:EccC:EccD:EccE, interacting in a 1:1:2:1 stoichiometry. EccC is a pivotal component of the pore complex, providing the secretion driving force via the hydrolysis of ATP (Famelis *et al.*, 2019 ▸; Poweleit *et al.*, 2019 ▸; Bunduc *et al.*, 2021 ▸; Beckham *et al.*, 2021 ▸). In the ESX-2 to ESX-5 systems, EccC consists of two N-terminal transmembrane (TM) helices connected to two additional helices (the stalk region) and a domain of unknown function (EccC^DUF^), which are followed by three FtsK/SpoIIIE AAA+ ATPase domains, here referred to as D1, D2 and D3 (EccC^D1–D3^; Famelis *et al.*, 2023 ▸; Bunduc *et al.*, 2021 ▸; Fig. 1 ▸ *b*). This multi-domain organization is also shared by EccC orthologues (EssC) found in *Firmicutes* (Mietrach *et al.*, 2020 ▸), which differ in ESX-1, where EccC is replaced by two functionally equivalent fragments: EccC_1_a, containing DUF and D1 domains, and EccC_1_b, containing D2 and D3 domains (Famelis *et al.*, 2023 ▸; Houben *et al.*, 2014 ▸). EccC/EssC enzymes are expected to operate through a mechanism involving their hexamerization. In line with this, the cytosolic region of *Mtb*EccC_5_ has been observed to transit from an extended/open state to a contracted/closed state in which the enzyme multimerizes to form the cytosolic chamber that is expected to accommodate the effectors to be secreted (Bunduc *et al.*, 2021 ▸; Fig. 1 ▸ *a*).

The D1–D3 domains exhibit the three-layer α–β–α core structure typically found in prokaryotic AAA+ enzymes, which is frequently followed by a C-terminal α-helical lid domain that is missing in EccC/EssC enzymes (Zoltner *et al.*, 2016 ▸). The hexameric architecture observed in the ESX-3 and ESX-5 complexes is in line with the functional oligomeric state frequently found in prokaryotic AAA+ enzymes. The latter include the FtsK and SpoIIIE ATPases, which are close relatives of EccC and are reported to function as hexamers, and in which the ATPase sites are located at the interface between two adjacent protomers (Miller & Enemark, 2016 ▸; Leipe *et al.*, 2002 ▸; Bunduc *et al.*, 2021 ▸; Rosenberg *et al.*, 2015 ▸). In such cases, both subunits provide the *cis*- and *trans*-acting catalytic elements required to form the active site. The former includes the Walker A motif, the Walker B motif and Sensor 1, which are typically located in the loop connecting β1 and α1, the C-terminus of β3 and the loop connecting β4 and α4, respectively. The Walker A motif, also referred to as the P-loop, consists of a glycine-rich sequence (G_1_ *xx*G_2_ *x*G_3_K[S/T] in FtsK homologues) that is involved in stabilization of the phosphate group of the nucleotide via highly conserved lysine and serine/threonine residues that enable the correct orientation of the γ-phosphate required for ATP hydrolysis (Miller & Enemark, 2016 ▸; Leipe *et al.*, 2002 ▸). The Walker B motif provides two acidic residues (*hhhh*DE in FtsK homologues, where *h* is any hydrophobic amino acid), the second of which acts as the catalytic base that activates the water involved in nucleophilic attack on the γ-phosphate (Miller & Enemark, 2016 ▸; Leipe *et al.*, 2002 ▸). Sensor 1 is a key catalytic element consisting of a polar residue, which is typically an asparagine but can also be a serine, threonine or aspartate. It is located between the Walker motifs to assist in the correct orientation of the catalytic glutamate towards the nucleophilic water. *Trans*-acting elements are positively charged amino acids, mostly (but not exclusively) arginine residues, and thus are referred to as arginine fingers or sensors (Miller & Enemark, 2016 ▸; Leipe *et al.*, 2002 ▸). The latter include the Arg finger, which consists of an arginine (or lysine) residue located at the end of the α4 helix that orients towards the neighbouring active site to form contacts with the γ-phosphate. Through this interaction, the Arg finger favours the transition state for hydrolysis, thus playing an important role in ATP hydrolysis and, in some cases, in enzyme multimerization (Miller & Enemark, 2016 ▸; Ogura *et al.*, 2004 ▸). The other two *trans*-acting motifs in AAA+ enzymes are Sensors 2 and 3, which are generally arginines that are involved in sensing and/or stabilizing the ATP/ADP-bound states and promoting conformational changes coupled to these states or nucleotide hydrolysis (Miller & Enemark, 2016 ▸; Li *et al.*, 2015 ▸).

The first structural and functional studies conducted by Rosenberg and coworkers on EccC from *Thermomonospora curvata* (*Tcr*EccC) showed that the D1 domain is an active ATPase that exhibits fully conserved Walker A (G*xx*G*x*GK[S/T]) and Walker B (*hhhh*DE) motifs with respect to FtsKs as a close canonical orthologue, including the Arg-finger *trans*-acting motif (Rosenberg *et al.*, 2015 ▸). This and subsequent studies on EccCs/EssCs from *Mtb* and *S. aureus* showed that the Walker A and B motifs are, however, degenerated in the D2 and D3 domains, which aligns with the nondetectable or poor catalytic activities observed for these domains, which have been proposed to play a regulatory role (Rosenberg *et al.*, 2015 ▸; Wang *et al.*, 2020 ▸; Zoltner *et al.*, 2016 ▸). More recently, structural characterization of the *Msm*ESX3 complex revealed that the N-terminal domain of unknown function (DUF) exhibits an ATPase-like fold, as also observed in the subsequently reported DUF domains of the *Mtb*ESX-5 and *Mxp*ESX-5 complexes (Famelis *et al.*, 2019 ▸; Bunduc *et al.*, 2021 ▸; Beckham *et al.*, 2021 ▸). The authors described the presence of a Walker B motif (*hhhh*DD^320^) in 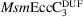 , which is partially conserved with respect to FtsK orthologs (*hhhh*DE), and how the mutation of each aspartate to alanine obliterates secretion. These observations highlight the essentiality of 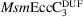 in secretion and point to a potential ATPase activity of this domain (Famelis *et al.*, 2019 ▸). The structure of 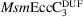 is however devoid of ATP, analogously to the 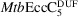 and 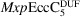 structures, which exhibit valine and isoleucine residues, respectively, that replace the catalytic amino acid (Famelis *et al.*, 2019 ▸; Bunduc *et al.*, 2021 ▸; Beckham *et al.*, 2021 ▸). These observations expose a variable degeneration of the Walker B motif across different EccC^DUF^ domains that may impair the ATPase activity to differing extents. In addition, the 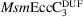 , 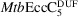 and 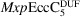 domain structures reveal a noncanonical arrangement of the Walker A motif lacking the typical P-loop structure, which is replaced by an extended α-helical conformation. Interestingly, this arrangement is also observed in the structure of EssC^D3^ from *S. aureus* (*Srs*EssC^D3^; PDB entry 6tv1), which is also devoid of ATP and exhibits an ATP-binding affinity in the low-millimolar range (Mietrach *et al.*, 2020 ▸).

The structural and functional information available for T7SSs leaves outstanding questions regarding the molecular basis underlying the ATPase activity proposed for the DUF domain. In this regard, here we report the crystallographic structure of the 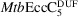 domain at 2.05 Å resolution, which provides an unambiguous model showing a nucleotide-free structure with degenerated *cis*-acting and *trans*-acting elements involved in ATP binding and hydrolysis. Our high-resolution structure, together with a biophysical assessment of the interaction of 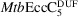 with ATP/Mg^2+^ *in vitro*, supports the absence of ATPase activity in this domain. These results are in line with an *in silico* analysis carried out in other EssC and EccC enzymes, which reveals the presence of degenerated DUF domains in other mycobacterial and non­mycobacterial T7S systems that are likely to exhibit null or deficient ATPase activity. These findings suggest that DUF domains play a different role in the secretion process that, based on an *in silico* model of *Mtb*EccC_5_ and the mutagenesis studies reported for *Msm*ESX3 (Famelis *et al.*, 2019 ▸), we propose it may be related to the aperture of the membrane-pore complex during the secretion process.

## 2. Materials and methods

### 2.1. Protein expression and purification

**Figure 2:**
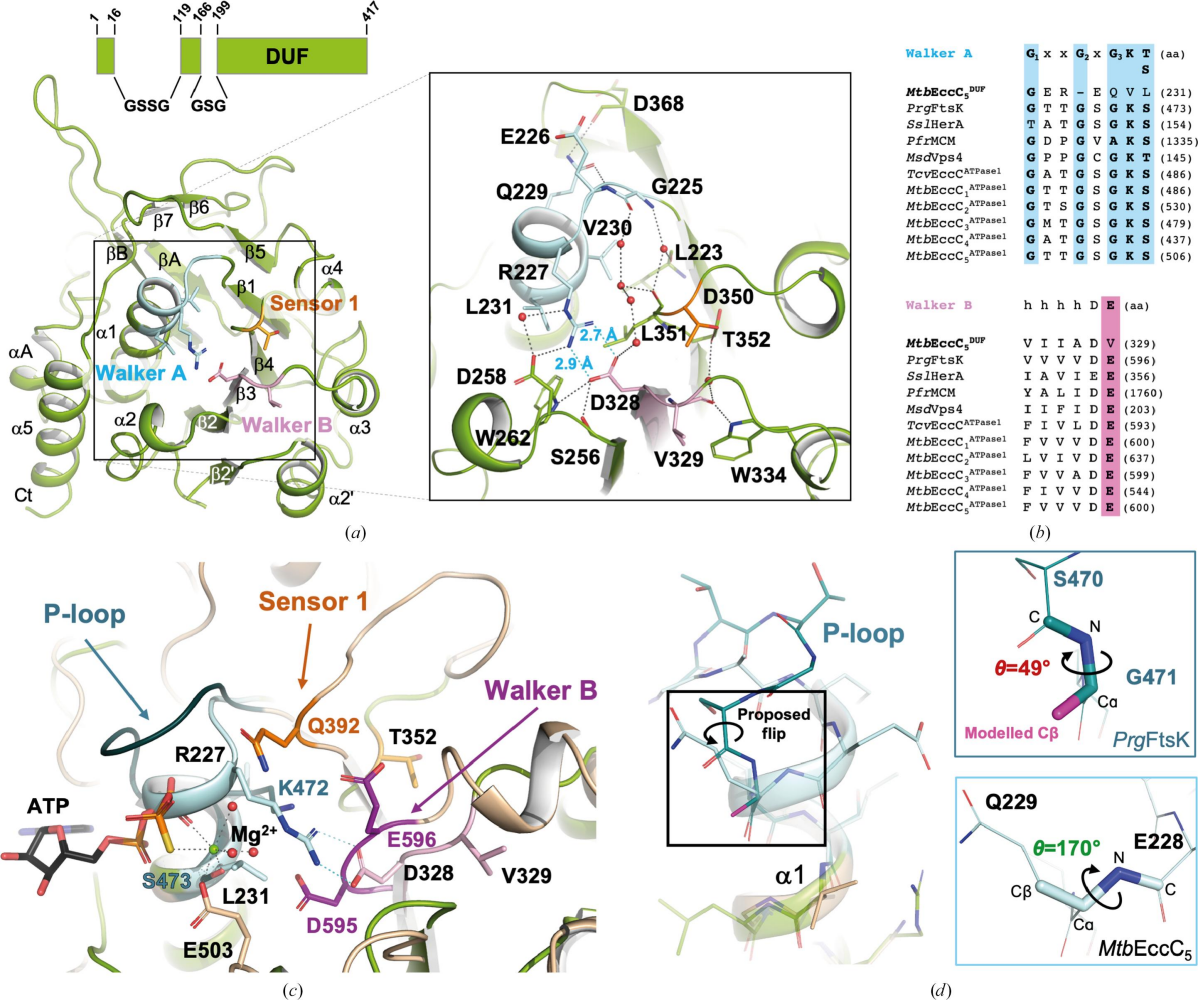
(*a*) A general view of the 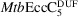 crystal structure represented as green cartoons is shown (left), as well as details of the putative ATP-binding site (right). Salt-bridge and hydrogen-bond interactions are indicated as dashed lines in blue and black, respectively, as well as the bond distances between Arg227 and Asp328. The Walker A, Walker B and Sensor 1 motifs are coloured blue, pink and orange, respectively, showing key residues (sticks) and water molecules (red spheres). A scheme with details of the regions of 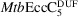 included in the crystallographic construct is shown. (*b*) Sequence alignment of the Walker A and Walker B motifs of 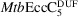 with the canonical ATPase domains of *Prg*FtsK, *Ssl*HerA, *Pfr*MCM, *Msd*Vps4 and 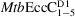 , showing the consensus sequence at the top. Key amino acids involved in nucleotide interaction/hydrolysis are highlighted in blue and pink, respectively. (*c*) Superimposition of the 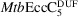 and *Prg*FtsK (PDB entry 4r7y, beige) structures represented as cartoons. The Walker A, Walker B and Sensor 1 motifs are coloured light blue, pink and orange, respectively, in 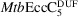 and in dark teal, purple and dark orange, respectively, in *Prg*FtsK. In *Prg*FtsK, the magnesium cation is represented as a green sphere, while ATP (in black) and residues key to the interaction with the nucleotide are represented as sticks. The Arg227–Asp328 salt bridge observed in 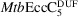 and the hydrogen bonds involved in ATP/Mg^2+^ stabilization in *Prg*FtsK are represented as dashed lines in blue and black, respectively. (*d*) Detail of the Walker A motif in 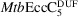 and *Prg*FtsK, showing the favourable dihedral angle C_*i*_—N_*i*+1_— 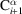 — 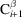 observed between Glu228 and Gln229 in the former compared with the nonfavourable dihedral angle generated between Ser470 and Gly471 of *Prg*FtsK when the glycine is substituted by an alanine.

A protein construct containing the DUF domain of EccC_5_ from *M. tuberculosis* ( 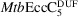 , residues 1–417; UniProt entry P9WNA5) was designed for recombinant production. Residues 17–118 and 167–198 were replaced by GSSG and GSG sequences, respectively, in order to remove the transmembrane (TM) and stalk regions and enable production as a soluble protein (see Fig. 2 ▸ and Supplementary Fig. S1). The construct contained an N-terminal 6×His tag followed by a SUMO tag and a PreScission 3C cleavage site. The DNA coding for the construct cloned into a pET-28a plasmid (between NcoI and XhoI restriction sites) was purchased from GenScript with codon optimization for *Escherichia coli* expression ( 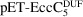 ). Expression of 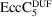 was performed in *E. coli* BL21 (DE3) Star (Invitrogen) cells using 2×TY medium supplemented with kanamycin (50 µg ml^−1^). The cells were grown at 37°C to an OD_600 nm_ of 0.8 and then cooled to 16°C for an hour. Expression was then induced at 16°C by the addition of 1 m*M* isopropyl β-d-1-thiogalactopyranoside for 20 h. The cells were harvested by centrifugation at 5251*g* for 20 min at 4°C and the cell pellets were stored at −20°C.

The cell pellets were thawed and resuspended in 20 m*M* Tris–HCl pH 7.5, 500 m*M* NaCl, 10 m*M* imidazole (buffer *A*) supplemented with 5 m*M* MgCl_2_, 1 m*M* MnCl_2_ and 1 µg ml^−1^ DNAse (Sigma). This step was performed at room temperature, while subsequent purification steps were carried out at 4°C. The cells were disrupted by sonication and the clarified extract was loaded into a 1 ml HisTrap HP column (Cytiva) previously equilibrated in buffer *A*. Protein samples were eluted using an imidazole gradient (from 10 to 500 m*M*) and then buffer-exchanged to 20 m*M* Tris–HCl pH 7.5, 150 m*M* NaCl (buffer *B*) using a PD-10 desalting column (Cytiva). Samples were diluted to 1 mg ml^−1^ in buffer *B* prior to overnight cleavage with 3C protease. The cleaved protein was concentrated using Amicon 10 kDa molecular-weight cutoff concentrators (Millipore) and further purified by size-exclusion chromatography (SEC) using a Superdex 200 (16/60) column (Cytiva) pre-equilibrated with buffer *B* or 20 m*M* HEPES pH 7.5, 150 m*M* NaCl, 5 m*M* MgCl_2_ (buffer *C*) for crystallographic or ITC experiments, respectively. Pure protein fractions obtained from SEC were pooled, concentrated and flash-frozen in liquid nitrogen for storage at −80°C. Protein sample purity and quantification were assessed by SDS–PAGE and UV–Vis absorbance at 280 nm, respectively.

### 2.2. Protein crystallization, structure resolution and analysis

All crystallization experiments were carried out using the sitting-drop vapour-diffusion method at 20°C in 96-well MRC plates (Hampton Research) employing an Oryx8 robot (Douglas Instruments). Initial crystals of 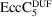 were obtained in condition F2 of the Index screen. After optimization, the best crystals grew in 300 nl droplets formed by mixing 150 nl protein solution at 10 mg ml^−1^ and 150 nl precipitant condition [25%(*w*/*v*) PEG MME 2K, 300 m*M* trimethylamine *N*-oxide, Tris–HCl pH 8.0]. The crystals were flash-cooled in liquid nitrogen without cryoprotection for X-ray data collection, which enabled the measurement of a complete X-ray data set to 2.05 Å resolution at 100 K using synchrotron radiation at ALBA, Barcelona, Spain. The data set was indexed and integrated with *XDS* (Kabsch, 2010 ▸) and scaled and reduced using *AIMLESS* (Evans & Murshudov, 2013 ▸). The crystals belonged to space group *P*1, with unit-cell parameters *a* = 48.30, *b* = 52.24, *c* = 57.44 Å, α = 88.39, β = 84.60, γ = 80.17° (Table 1 ▸). Structure solution was carried out using the molecular-replacement method using *Phaser* (McCoy *et al.*, 2007 ▸) with the coordinates of the 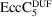 structure (residues 1–416) solved by cryo-EM (PDB entry 7npt; Bunduc *et al.*, 2021 ▸) as the search model. A single and unambiguous solution was obtained, which consisted of two 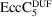 molecules in the asymmetric unit. The initial model was subjected to alternate cycles of model building with *Coot* (Emsley *et al.*, 2010 ▸) and refinement with *Phenix* (Liebschner *et al.*, 2019 ▸) and *BUSTER* (Bricogne *et al.*, 2017 ▸) using NCS restrictions. The electron-density maps allowed us to model both molecules of the asymmetric unit almost completely along with 287 water molecules. Residues 1–16-GSSG-119–123 (in chains *A* and *B*), 275–283 and 311–315 (in chain *A*) and 274–283 (in chain *B*) were not modelled due to poor electron density being observed for these residues. The geometry of the final model was validated using *MolProbity* (Chen *et al.*, 2010 ▸). Dihedral angles were analysed with *Coot* (Emsley *et al.*, 2010 ▸) and *Mogul* (version 2020.3.0). Figures were generated using *PyMOL* (version 2.0; Schrödinger). The atomic coordinates and structure factors have been deposited in the Protein Data Bank under accession code 8rin.

### 2.3. Differential scanning fluorimetry

Differential scanning fluorimetry (DSF) assays were carried out to estimate the inflection temperature (*T*_i_) associated with unfolding transitions of the 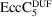 construct in the absence and presence of nucleotide/Mg^2+^. The experiments consisted of monitoring the variation in the emission of fluorescence by buried and exposed tryptophan residues at wavelengths of 350 and 330 nm, respectively, using a Tycho NT.6 (NanoTemper). DSF experiments were set up in a final volume of 10 µl buffer *C* containing the 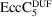 construct at 2 µ*M* in the absence or presence of ADP–AlF_3_ at 4 m*M*. Mixtures of protein with the ATP analogue were incubated for 15 min before DSF measurements. Graph representations and analysis were performed using the Tycho analysis interface. Three independent runs were used in each case to calculate mean *T*_i_ values and the corresponding standard deviations.

### 2.4. Isothermal titration calorimetry

The ATP-binding and ATPase activities of the 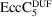 domain were assessed by isothermal titration calorimetry (ITC) using an ITC-VP instrument from GE Instruments. 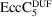 at 61 µ*M* was loaded into the cell at 25°C and was titrated with ATP at 1.3 m*M* (in the syringe) using 15 injections, firstly of 1 µl, then of 10 µl and subsequently of 20 µl at intervals of 5 min. Both protein and ATP were prepared in buffer *C*. Analysis of the results was carried out and final figures were generated using the *Origin* 7 ITC software.

### 2.5. Primary-, secondary- and tertiary-structure analysis *in silico*

A comparative analysis of the putative ATP/Mg^2+^-binding site across DUF domains and comparison with canonical ATPases was performed by *Clustal Omega* multi-sequence alignment (Sievers *et al.*, 2011 ▸) and by structure superimposition in *PyMOL* (version 2.0; Schrödinger) using experimental coordinates deposited in the Protein Data Bank and models predicted by *AlphaFold* (Jumper *et al.*, 2021 ▸) available at UniProt. The ATPases used in sequence alignment correspond to FtsK from *Pseudomonas aeruginosa* (*Prg*FtsK; UniProt entry Q9I0M3; PDB entry 2iut; Massey *et al.*, 2006 ▸), HerA DNA translocase from *Sulfolobus solfataricus* (*Ssl*HerA; UniProt entry Q97WG8; PDB entry 4d2i; Rzechorzek *et al.*, 2014 ▸), MCM helicase from *Pyrococcus furiosus* (*Pfr*MCM; UniProt entry Q8U3I4; PDB entry 4r7y; Miller *et al.*, 2014 ▸), Vps4 from *Metallosphera sedula* (*Msd*Vps4; UniProt entry A4YHC5; PDB entry 4d81; Caillat *et al.*, 2015 ▸), EccC from *Thermomonospora curvata* (*Tcr*EccC; UniProt entry D1A4G7), *Mtb*EccCa_1_ (UniProt entry P9WNB3), *Mtb*EccC_2_ (UniProt entry O05450), *Mtb*EccC_3_ (UniProt entry P9WNA9), *Mtb*EccC_4_ (UniProt entry P9WNA7), *Mtb*EccC_5_ (UniProt entry P9WNA5; PDB entries 7npt and 7npr; Bunduc *et al.*, 2021 ▸), 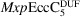 (UniProt entry I0RZI0; PDB entry 7b9s; Beckham *et al.*, 2021 ▸), *Msm*EccCa_1_ (UniProt entry A0A653FCM4), *Msm*EccC_3_ (UniProt entry A0QQ40; PDB entry 6sgx; Famelis *et al.*, 2019 ▸), *Msm*EccC_4_ (UniProt entry A0QSN0), EccC from *Geobacillus thermodenitrificans* (*Gth*EccC; UniProt entry A4IKE7), EssC from *Staphylococcus aureus* (*Srs*EssC; UniProt entry Q932J9; PDB entry 6tv1), EssC from *Clostridium* sp. (*Csp*EssC; UniProt entry A0A3R6STG9), EssC from *Streptococcus oralis* (*Srl*EssC; UniProt entry A0A656Z0M6) and YukB from *Bacillus subtilis* (*Bsb*YukB; UniProt entry C0SPA7). Structural analysis of the dihedral angles and their occurrence in the Cambridge Crystallographic Data Centre (CCDC) database was performed using *Mogul* (version 2020.3.0). *AlphaFold*2-*multimer* (Jumper *et al.*, 2021 ▸; Evans *et al.*, 2021 ▸) was employed to explore potential protein–protein interactions of 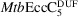 with the *Mtb*PE25–PPE41 effector complex (UniProt entries I6X486 and Q79FE1, respectively; Abdallah *et al.*, 2006 ▸; Daleke *et al.*, 2012 ▸), inter-protomer interactions of 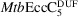 with the ubiquitin-like domain of 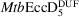 (UniProt entry P9WNP9) and the potential hexamerization of 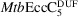 . Feasible predicted interfaces were analysed using *jsPISA* (Krissinel, 2015 ▸) in order to identify inter-monomer/domain contacts, which were subsequently inspected and validated in *PyMOL*. *jsPISA* was also used to analyse the predicted interfaces using the interaction radar score, which estimates the likelihood of a biological ensemble based on a statistical analysis of all interfaces found in the PDB. The interaction radar is divided into probability circles (0–100%). The larger the area, the higher the probability of the interface having biological significance, and area values fitting or above the 50% probability circle are likely to be part of a biological ensemble (Krissinel, 2015 ▸).

## 3. Results and discussion

### 3.1. High-resolution structure of the EccC_5_^DUF^ domain

The overall crystallographic structure of the 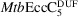 domain (Fig. 2 ▸ *a*) consists of an α–β–α sandwich that is highly homologous to the α/β fold found in the core of AAA+ domains. The core structure consists of a six-stranded parallel β-sheet (β5–β1–β4–β3–β2–β2′) further extended by an additional two-stranded antiparallel sheet (β6–β7). This β-sheet core is flanked by two helices (α1 and α2) on one side and four helices (α2′A, α2′B, α3 and α4) on the other side. The C-terminal α-helical lid present in other prokaryotic AAA+ domains is missing in the 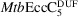 structure, which is flanked by two parallel helices (αA and αB) and a two-stranded antiparallel sheet (βA–βB) that connects to the stalk domain. This structure is conserved in both 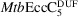 molecules present in the asymmetric unit (r.m.s.d. of 0.173 Å for 218 C^α^ atoms). Superimposition of the crystallographic and  cryo-EM ( 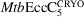 ; PDB entry 7npr; Bunduc *et al.*, 2021 ▸) models shows overall structure conservation (r.m.s.d. of 0.671 Å for 198 C^α^ atoms), with the only significant differences in the N-terminal region (residues 1–16 are not defined in the electron-density maps). However, despite the overall conservation of the fold, other differences between our structure and 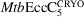 are found, as will be described below.

The proposed ATP-binding site should be located at the N-terminus of the α1 helix and the C-terminus of the β3 strand, which are expected to contain the P-loop and the Walker B motif, respectively. Sequence analysis of the Walker A motif shows a highly degenerated and one amino acid shorter sequence (GEREQVL231) that is missing the conserved G_2_ and G_3_ glycines as well as the Lys and Thr/Ser residues that are key for ATP/Mg^2+^ stabilization (Val30 and Leu231 in 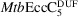 ; Fig. 2 ▸ *b*). Inspection of the 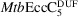 crystal model shows an ATP-free structure in which the degenerated Walker A motif folds as an extended α1 helix instead of the standard P-loop conformation. In this arrangement, α1 would clash with the nucleotide molecule, as revealed by structural superimposition of 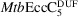 with FtsK from *P. aeruginosa* (*Prg*FtsK), a closely homologous canonical ATPase. To understand the structural basis of this extended α1 structure, we investigated the impact of G_2_ and G_3_ variations in 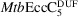 , as invariant flexible features involved in both turns of the P-loop motif. To do so, we modelled the replacement of G_3_ by alanine in *Prg*FtsK (G471A) *in silico*, which shows how the newly added C^β^ gives rise to an unfavoured dihedral angle C_*i*_—N_*i*+1_— 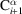 — 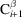 (θ = 49°) between Ser470 and Ala471 (Fig. 2 ▸ *d*). This is supported by the low occurrence of this torsion angle when searching the CCDC database (Supplementary Fig. S1). This disfavoured conformation is prevented in 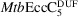 by a flip of the Glu228-Gln229 peptide bond, leading to the extended α-helical structure observed in α1 (Fig. 2 ▸ *d*). Analogously, the absence of G_2_ should foster an α-helical conformation to avoid un­favourable dihedral angles between the two adjacent non­glycine amino acids. Altogether, this points to variations of G_2_ and G_3_ as structural factors responsible for the extended α1 helix observed in 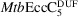 . Inspection of the Walker B motif also shows conformational deviations in 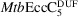 , which include a shorter β3–α3 loop and a hydrophobic residue (Val329) replacing the catalytic base (Fig. 2 ▸ *a*). Val329 rests partially buried between the β3–α3 and β4–α4 loops in a conformation that is well stabilized by a hydrogen-bond network with nearby residues. This drags the Walker B motif away from the P-loop by >2 Å compared with *Prg*FtsK, thus providing the room necessary to accommodate the extended α1 helix. This noncanonical arrangement of the Walker motifs is further stabilized by numerous direct and water-mediated interactions. These include a hydrogen bond between the side-chain amide group of Gln229 and the main-chain N atom of Glu226 and the formation of a salt bridge between the side chains of Arg227 and Asp328, which stabilize the extended α1 structure and are well defined in the electron-density maps (Supplementary Fig. S1). Moreover, the short bond distances measured for the salt-bridge interaction indicate that this is a strong contact that, overall, should further challenge the structural rearrangement of this nonproductive conformation observed in α1 for ATP binding. Superimposition of the crystal and cryo-EM structures of *Mtb*EccC_5_ shows overall conservation of the noncanonical arrangement observed in the Walker motifs in both models (Supplementary Fig. S1). However, significant differences were found in the structural features that shape the architecture of the degenerated ATP-binding site, supported by the electron-density and cryo-EM maps (Supplementary Fig. S1). These comprise many direct or water-mediated hydrogen bonds that stabilize the Walker and Sensor 1 motifs, including an interaction between Gln229 and Glu226 that is too long to form a hydrogen bond in 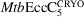 and a water-mediated contact between the side chains of Arg227 and Asp258, which seems to assist the side chain of Arg227 in adopting an optimal orientation to interact with Asp328 and was not observed in the cryo-EM model. Moreover, in contrast to 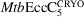 , the crystal structure of 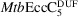 shows a bidentate salt bridge between the side chains of Arg27 and Asp328 that is well defined in the electron-density maps and contributes to optimize the interactions that stabilize the non­canonical configuration of the Walker A motif (Supplementary Fig. S1). Thus, our structure provides an unambiguous model defining the noncanonical ATP-binding site of *Mtb*EccC_5_ at high resolution, which has allowed us to acquire a detailed description and understanding of the key structural features contributing to its degenerated arrangement.

We next analysed the regions expected to contain the *trans*-acting elements in 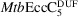 (Supplementary Fig. S1). No basic residues were observed at the end of α4 as candidates to act as an Arg finger. Only Arg362 was found in the vicinity, and was further down the α4 sequence. However, its quite buried position in the structure makes it a poor candidate to function as an Arg finger. The helical C-terminal lid, which is habitually found in prokaryotic AAA+ clades and should contain Sensor 2, is missing in 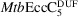 . Besides, inspection of the α3 helix shows no basic residues in this region to form a potential Sensor 3. Taking all these observations together, our structural analysis of 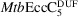 shows the degeneration and absence of *cis*- and *trans*-acting elements, respectively, in 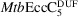 , which would support the lack of ATPase activity in this domain. These observations are in line with our failed attempts to co-crystallize 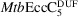 with ATP/Mg^2+^, with structures devoid of nucleotide and magnesium instead being obtained.

### 3.2. ATP-binding and ATP-hydrolysis analysis

**Figure 3:**
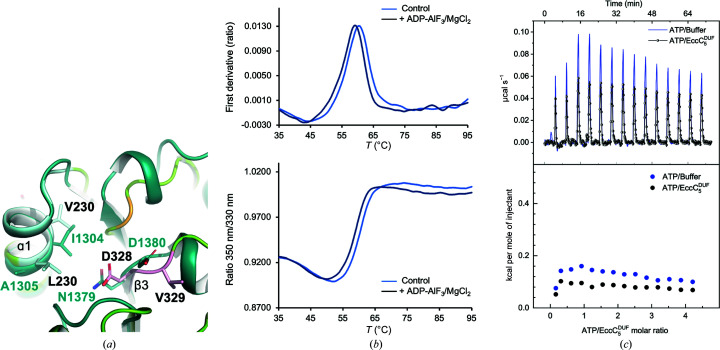
(*a*) Superimposition of the 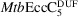 (green) and *Srs*EssC^D3^ (PDB entry 6vt1, dark blue) crystal structures showing details of their degenerated ATP-binding sites. Residues occupying key positions for nucleotide/magnesium interaction and hydrolysis are labelled and represented as sticks. The Walker A, Walker B and Sensor 1 motifs of 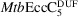 are coloured following the same colour code as in Fig. 2 ▸. (*b*) Superimposition of 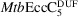 DSF profiles in the absence (control) and presence of ADP–AlF_3_ (4 m*M*) and Mg^2+^ (5 m*M*). The failure of ADP–AlF_3_/Mg^2+^ to stabilize the domain structure against thermal denaturation was consistent with a lack of nucleotide binding. (*c*) ITC titration of ATP (1.3 m*M*) into 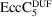 (60 µ*M*) and buffer *C* (black and blue data, respectively). The upper and lower panels show raw thermograms and normalized heat effect per mole of ATP injected versus ATP: 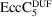 molar ratio, respectively.

The structural arrangement of the putative ATP-binding site of 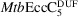 resembles the nucleotide-binding pocket observed in the structure of the *Srs*EssC^D3^ domain (PDB entry 6tv1), which has been reported to bind ATP with an affinity in the low-millimolar range (Mietrach *et al.*, 2020 ▸; Fig. 3 ▸ *a*). This poor but detectable binding points to potential structural rearrangements of the ATP-binding site of *Srs*EssC^D3^ that enable interaction with the nucleotide. Therefore, we analysed the interaction of 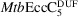 with ATP/Mg^2+^ in order to explore a possible reorganization of the ATP-binding site that might enable nucleotide binding and hydrolysis in solution using two different biophysical approaches.

Firstly, the interaction of 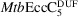 with ADP–AlF_3_ (used as an ATP analogue) was assessed by DSF in the presence of magnesium. Despite the high ADP–AlF_3_/ 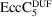 concentration ratio used (∼4 m*M*/2 µ*M*), the experiments showed that the difference between *T*_i_ values in the presence (59.13 ± 0.04°C) and absence (60.46 ± 0.04°C) of nucleotide was slightly negative (around −1.3°C) (Fig. 3 ▸ *b*) and very close to the error limit of the device, thus pointing to an absence of binding. Nucleotide interaction and ATP hydrolysis were next assessed by ITC in the presence of magnesium. The heat released upon the titration of ATP into 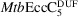 was comparable to the thermal effect of ATP dilution in the range of concentrations tested (Fig. 3 ▸ *c*). Additionally, no thermal power deflection (proportional to the potential substrate concentration in the cell) was observed after each ATP injection, as would be expected if the nucleotide was being hydrolysed (Menéndez, 2020 ▸). Overall, the ITC and DSF results support the lack of ATPase activity of 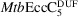 .

### 3.3. The nucleotide-binding site in other EccC^DUF^ domains

**Figure 4:**
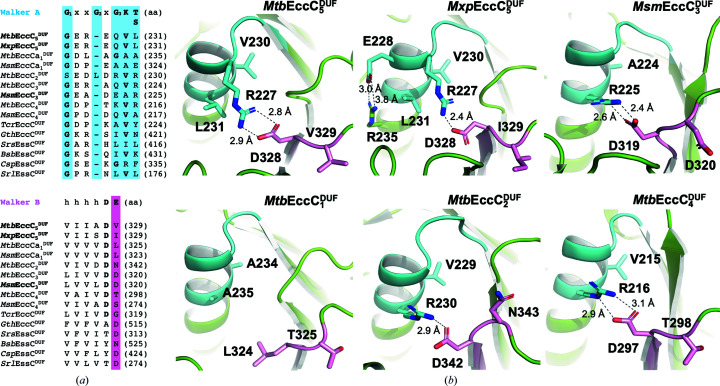
Analysis of a putative ATP/Mg^2+^-binding site in EccC^DUF^ domains of ESX-1–ESX-5 systems from *Mtb*, *Msm* and *Mxp*. (*a*) Sequence alignment of the Walker A and B motifs (coloured blue and pink, respectively). (*b*) Structural details of the Walker A and B motifs observed in 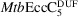 (crystal structure), 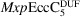 (PDB entry 7b9s), 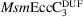 (PDB entry 6gsx), 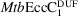 (*AlphaFold* model, UniProt entry P9WNB3), 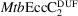 (*AlphaFold* model, UniProt entry O05450) and 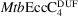 (*AlphaFold* model, UniProt entry P9WNA7) represented as cartoons and following the same colour code as in (*a*). Amino acids substituting key residues for ATP/Mg^2+^ interaction, or involved in hydrogen-bond/salt-bridge interactions, are depicted as sticks.

Given the structural degeneration that was observed in the 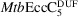 structure, and its observed inability to interact with ATP using X-ray crystallography, DSF and ITC, we decided to examine the putative ATP-binding site in other DUF domains. With this purpose, homologous EccC^DUF^ domains and orthologous EssC^DUF^ domains from mycobacterial and nonmycobacterial species were used in sequence alignment (Fig. 4 ▸ *a*), which showed degeneration of the Walker A motif in all of the domains inspected. Analogously to 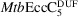 , these motifs present a sequence that is one amino acid shorter, in which the conserved residues G_2_ and G_3_ are missing and substituted by a nonglycine amino acid, respectively. They also lack both the lysine and threonine/serine residues that are required for interaction with ATP/Mg^2+^. The DUF domains inspected also exhibit degeneration of their Walker B motifs, in which the catalytic glutamate is replaced by other hydrophobic or polar amino acids (Rosenberg *et al.*, 2015 ▸; Zoltner *et al.*, 2016 ▸; Wang *et al.*, 2020 ▸). Interestingly, analysis of the 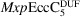 and 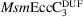 structures showed that their Walker motifs adopt a similar arrangement to that observed in 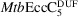 , including an extended α1 helix (Fig. 4 ▸ *a*). Analogously to 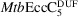 , the extended α1 helix can be explained by the amino-acid variations observed at the G_2_ and G_3_ positions with respect to canonical ATPases, which should elicit a helical conformation compared with the P-loop structure. This structural reorganization was also found in the structural models of 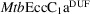 , 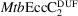 and 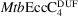 and in six other DUF domain structures predicted by *AlphaFold*, all of which present the same extended conformation of α1 (Fig. 4 ▸ and Supplementary Fig. S2). Inspection of the Walker motifs in the 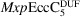 and 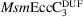 structures solved by cryo-EM also reveal an arginine–aspartate salt bridge connecting α1 to the C-terminus of β3 (Fig. 4 ▸ *b*). The salt bridge observed in 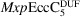 involves an arginine at the N-terminus of α1, analogously to as in 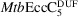 , while the salt bridge in 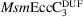 is formed by an arginine substituting the canonical threonine/serine of the Walker A motif (Fig. 4 ▸ *a*). Of the 15 sequences analysed, five mycobacterial DUF domains exhibit an arginine/lysine and aspartate that could potentially form a salt bridge, which is also predicted in the *AlphaFold* models of 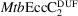 and 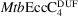 , where it is likely to contribute to stabilize this non­productive arrangement for ATP-Mg^2+^ recognition (Fig. 4 ▸ *b*). Thus, our analysis shows that the noncanonical structure observed in the 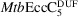 Walker motifs is present in the cryo-EM structures of 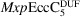 and 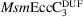 , as well as in the predicted models of other DUFs from T7Sa and T7Sb systems. Together, these observations support the idea that the DUF domains are degenerated ATPase domains that exhibit a nonfunctional structure for ATP binding and thus a poor or null ATPase activity.

### 3.4. Hexameric model of the N-terminal region of *Mtb*EccC_5_

**Figure 5:**
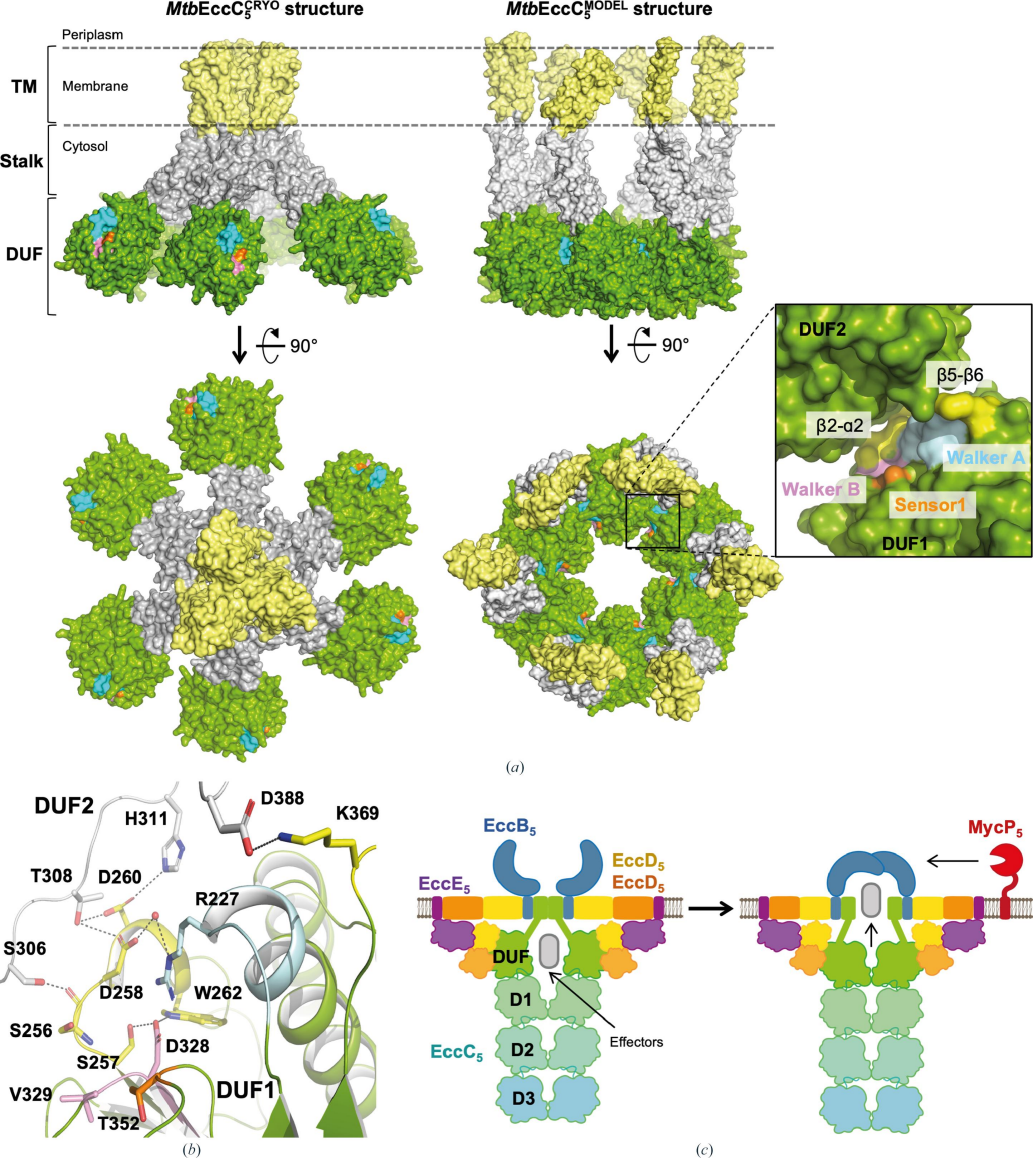
(*a*) Structural comparison of the EccC_5_ arrangement observed in the *Mtb*ESX-5 structure (PDB entry 7npr) with our model of the N-terminal region of *Mtb*EccC_5_ multimerizing as a hexamer. A surface representation of *Mtb*EccC_5_ molecules is shown, with the transmembrane, stalk and DUF domains coloured yellow, grey and green, respectively. The Walker A, Walker B and Sensor 1 motifs are coloured cyan, pink and orange, respectively. An enlarged view showing the molecular interface between adjacent DUF monomers in our model is shown in the box on the right, where identified contact regions are highlighted in yellow. (*b*) Details of the interface between adjacent DUF1 (grey) and DUF2 [using the colour code used in (*a*)] molecules are shown, with residues establishing contacts between the monomers and the Walker A and B motifs represented as sticks; their corresponding interactions are indicated as dashed lines. Water molecules are represented as red spheres. (*c*) Model showing the aperture of the TM region of *Mtb*EccC_5_ associated with the proposed multimerization of 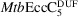 , in which this domain would transition from a non-multimerized form to a hexameric state to enable opening of the membrane pore.

The lack of ATPase function of EccC^DUF^ domains proposed here raises important questions about their role in the secretion process. The relevance of these questions is particularly stressed by site-directed mutagenesis studies conducted on *Msm*ESX-3, in which mutations of both of the aspartate residues of the Walker B motif of 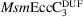 to alanines (D319A and D320A) were shown to abrogate secretion (Famelis *et al.*, 2019 ▸). This observation leads us to speculate that these mutations might either prevent proper folding of 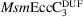 or affect protein–protein interactions that are key for secretion. Plausible DUF interactors might consist of PE/PPE effector proteins and other cytosolic ESX domains. Considering the second scenario, the ubiquitin-like domains of EccD (ULD or EccD^ULD^) and DUFs of neighbouring protomers emerge as reasonable inter-protomer interactors, given their proximity in ESX complexes. Given the conservation of the ATPase-like fold of DUF, it is conceivable to expect this domain to have retained the ability to multimerize in the context of DUF–DUF interplay, as has been observed for FtsK homologues and proposed for EccC^D1–D3^ domains (Rosenberg *et al.*, 2015 ▸; Miller & Enemark, 2016 ▸). This hypothesis aligns with the size-exclusion chromatography profile observed during the production of 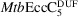 , which indicates the presence of monomeric, trimeric and hexameric species (Supplementary Fig. S3). We thus attempted to explore the interaction of 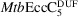 with the *Mtb*PE25–PP41 effector complex, as well as 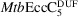 – 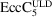 and 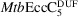 – 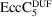 inter-protomer interactions, using *AlphaFold*. However, the predicted models showed unreliable and/or unfeasible interfaces (see the supporting information). This could be due to a genuine absence of these interactions, or to limitations in the modelling process arising either from the reliability of the theoretical model itself or the requirement for other domains/components of the cytosolic complex that were missing. Given these results, we decided to explore the potential multimerization of 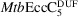 based on the experimental structures available of AAA+ enzymes in oligomeric states. To do so, we used the crystal coordinates of *Prg*FtsK in the hexameric state (PDB entry 2iuu) as a template to superimpose onto the N-terminal region of *Mtb*EccC_5_, including the TM, stalk and DUF domains, using the crystal coordinates of 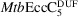 and 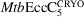 (PDB entry 7npr). This resulted in an hexameric model in which the 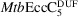 domains form a closed ring, with the stalk and TM regions located perpendicular to the DUF domains (Fig. 5 ▸ *a*). Analysis of the interface between adjacent DUFs shows that the Walker A, Walker B and Sensor 1 motifs are located near the neighbouring monomer, but they do not participate in inter-domain contacts (Fig. 5 ▸ *b*). However, direct interactions via residues in the β2–α2 loop, α2 and β5–β6 loop regions were identified. Interestingly, the β2–α2 region is located adjacent to the Walker A and B motifs, with which it forms direct interactions through several residues that include Arg227 and Asp328 (Fig. 5 ▸ *b*). Based on these interactions, mutations in Arg227 and Asp328 and/or other amino acids of both Walker motifs might impact on the structure of the β2–α2 region and, by extension, on 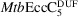 multimerization. This would also explain the lack of secretion observed in both Walker B motif mutants, D319A and D320A, of 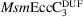 (Famelis *et al.*, 2019 ▸), which correspond to Walker B motif residues Asp328 and Val329 in 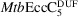 , respectively. Moreover, the residues identified at the DUF–DUF interface differ from those observed in the interaction with the 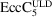 domain (Bunduc *et al.*, 2021 ▸), which shows the hexameric model of 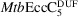 to be compatible with 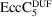 – 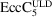 interplay as a key structural element in ESX complexes (Famelis *et al.*, 2019 ▸; Bunduc *et al.*, 2021 ▸; Beckham *et al.*, 2021 ▸). With regard to the TM and stalk regions, these are located perpendicularly and at the edge of the ring formed by 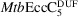 domains, with the TM regions of opposite monomers separated by a distance of ∼86 Å. This disposition notably contrasts with the EccC_3_ and EccC_5_ organization observed in the *Msm*ESX-3, *Mtb*ESX-5 and *Mxp*ESX-5 structures, in which the TM regions multimerize to form a helical bundle that connects, via the stalk region, to DUF domains positioned away from each other (Famelis *et al.*, 2019 ▸; Bunduc *et al.*, 2021 ▸; Beckham *et al.*, 2021 ▸; Fig. 5 ▸ *c*). Of note, the TM helical bundle is located at the centre of the membrane region, where it contributes to a close conformation of the membrane pore. Thus, the aperture of the TM and stalk regions depicted by our model suggests a potential conformational change of the N-terminal region of 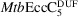 that would explain how the membrane pore might open through hexamerization of the DUF domain (Fig. 5 ▸ *c*). Given the distance measured across DUF domains in the 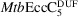 model (∼35 Å), and the dimensions of the periplasmic chamber observed in the *Mtb*ESX-5 complex (Bunduc *et al.*, 2021 ▸), the proposed hexamerization could allow a pore opening with a diameter of 35–45 Å, which would be capable of accommodating the secreted PE–PPE heterodimers. Overall, our *Mtb*EccC_5_ model suggests that DUF hexamerization is a plausible event that might play a critical role in the aperture of the membrane pore. This multimerization would be triggered by the interplay of DUFs with the EccC^D1–D3^ ATPase domains, thus linking ATP hydrolysis and effector recognition to the opening of the membrane pore necessary for secretion. Certainly, the inter-protomer interplay proposed here for DUF domains constitutes a hypothesis that requires further studies to be verified, while raising additional outstanding questions that require attention. For instance, how does the coupling of ATPase hydrolysis, effector recognition and multimerization of the D1–D3 ATPase domains occur with the proposed DUF–DUF and/or DUF–ULD interplay and the aperture of the membrane pore?

## 4. Conclusions

Here, we report the crystallographic structure of the 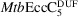 domain at high resolution, which reveals an ATP/Mg^2+^-free structure with both high degeneration and the absence of the *cis*- and *trans*-acting motifs required for the binding and hydrolysis of ATP, respectively. Among the most remarkable features, we find the absence of the catalytic glutamate required for nucleophilic attack on ATP and a noncanonical fold of the Walker A motif. Instead of the typical P-loop conformation, the Walker A motif adopts an extended α-helical arrangement that would clash with the nucleotide. Although the overall conservation of this arrangement is also observed in 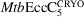 , our crystal structure provides previously unseen and unambiguously defined interactions that shape the observed noncanonical ATP-binding site. Moreover, our structural analysis of 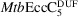 has led us to identify the structural features that underlie this arrangement. These include the variations of G_2_ and G_3_ observed in the Walker A motif of 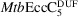 , which introduce geometrical restraints that favour the extended α1 helical structure instead of the canonical P-loop conformation. We also demonstrated the lack of interaction of 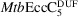 with the nucleotide in solution using DSF and ITC, which aligns with our futile attempts to co-crystallize this domain with ATP/Mg^2+^ and further supports the inability of the DUF domain to bind ATP. Our study also shows that the degenerated ATP-binding site observed in 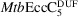 is present in the experimental and predicted structures of other homologous domains from T7Sa and T7Sb systems, which supports the notion that DUFs are degenerated ATPase domains that are nonfunctional in nucleotide interaction/hydrolysis. DUF domains emerge, however, as pivotal elements in the secretion process, as illustrated by mutagenesis studies of the Walker B motif of 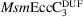 that led to the abrogation of *Msm*ESX-3 secretion. Putting all of these observations together, we speculate on the role of DUFs as structural elements involved in inter-protomer interplay, including a possible hexamerization of this domain with potential implications for the opening of the membrane pore during the secretion process. This hypothesis, which requires further studies, opens new important questions such as how ATPase hydrolysis, effector recognition and multimerization of the D1–D3 ATPase domains would couple with the proposed hexamerization of DUF domains and the aperture of the membrane pore.

## Supplementary Material

PDB reference: DUF domain of EccC_5_ from *Mycobacterium tuberculosis*, 8rin

Supporting information. DOI: 10.1107/S2059798324004248/jb5063sup1.pdf

## Figures and Tables

**Table 1:** X-ray crystallographic statistics for 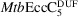 Values in parentheses are for the highest resolution shell.

Data-collection statistics	
Space group	*P*1
No. of complexes in asymmetric unit	2
*a*, *b*, *c* (Å)	48.30, 52.24, 57.44
α, β, γ (°)	88.39, 84.60, 80.17
Wavelength (Å)	0.9792
Beamline	BL13-XALOC
Data-collection temperature (K)	100
Resolution range (Å)	57.18–2.05 (2.11–2.05)
*R*_merge_ †	0.055 (0.354)
*R*_p.i.m._ ‡	0.035 (0.242)
Total No. of reflections	114509 (6654)
No. of unique reflections	32869 (2122)
Mean *I*/σ(*I*)	9.6 (2.6)
CC_1/2_	0.998 (0.869)
Completeness (%)	95.1 (77.9)
Multiplicity	3.5 (3.1)
Wilson *B* factor (Å^2^)	29.5
Refinement	
No. of atoms	
Total	4219
Protein	3932
Water	287
*R*_work_ (%)	0.19
*R*_free_ (%)	0.22
〈*B*〉 (Å^2^)	34.59
R.m.s.d., bond lengths (Å)	0.008
R.m.s.d., angles (°)	0.88

† *R*_merge_ = 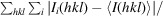 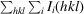 .

‡ *R*_p.i.m._ = 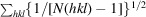 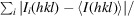 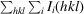 .
