# Supplementary material for: New insights into the domain of unknown function (DUF) of EccC5, the pivotal ATPase providing the secretion driving force to the ESX-5 secretion system
Source: Acta Crystallogr D Struct Biol. 2024 May 28;80(Pt 6):397–409. doi: 10.1107/S2059798324004248 (PMC11154593; doi:10.1107/S2059798324004248)
Supplement: Supplementary file 1 [file d-80-00397-sup1.pdf]

STRUCTURAL BIOLOGY

**Volume 80 (2024)**

**Supporting information for article:**

# New insights into the domain of unknown function DUF of EccC5, the pivotal ATPase providing the secretion driving force to the ESX-5 secretion system

**Fernando Ceballos-Zúñiga, Margarita Menéndez and Inmaculada Pérez-Dorado**

**S1. Prediction of protein:protein interactions using AlphaFold**

We employed AlphaFold2-multimer to explore on the one hand, the potential interaction of *Mtb*EccC$_5$$^{DUF}$ with the *Mtb*PE25:PPE41 complex as model effector proteins of *Mtb*ESX-5 (Abdallah *et al*., 2006; Daleke *et al*., 2012). On the other hand, we investigated the potential inter-protomer interactions mediated by the *Mtb*EccC$_5$$^{DUF}$ domain with the ubiquitin-like domain (ULD) of *Mtb*EccD$_5$ (*Mtb*EccD$_5$$^{ULD}$) as well as *Mtb*EccC$_5$$^{DUF}$ hexamerisation. To do so, the sequence corresponding to DUF, stalk and TM regions of *Mtb*EccC$_5$ (residues 1-417) was initially employed to model these interactions as summarised in table S1. For *Mtb*EccC$_5$:EccD$_5$$^{ULD}$ interaction, a prediction using a single copy of *Mtb*EccC$_5$ and two copies of *Mtb*EccD$_5$$^{ULD}$ was carried out. This prediction aimed to assess the capability of AF to predict the two intra-protomer DUF:ULD interfaces, experimentally observed in the cryo-EM structure of *Mtb*ESX-5 (Bunduc *et al*., 2021), ahead of calculating inter-protomer interactions. In all cases, AF models showed good structural predictions for the individual proteins/monomers (rmsd < 1 Å) when compared with the experimental structures: the *Mtb*EccC$_5$$^{DUF}$ crystal coordinates here reported, the crystallographic complex of *Mtb*PE25:PPE41 (PDB entry 4W4K), and *Mtb*EccD$_5$$^{ULD}$ determined by cryo-EM (PDB entry 7NPT). The predicted models calculated with effectors and *Mtb*EccD$_5$$^{ULD}$ showed ensembles non-compatible with ESX-5 architecture; and those calculated using six copies of *Mtb*EccC$_5$ yielded ensembles hexamerising through the TM region exclusively (see Table S1). To prevent interactions *via* stalk and TM domains, AF predictions were carried out using a *Mtb*EccC$_5$$^{DUF}$ sequence based on the crystallographic structure here reported (see Table S1). As before, all the models exhibited good structural predictions for the individual proteins/monomers (rmsd < 1 Å) when compared with the experimental structures. In this case, two of the calculated models with effectors revealed *Mtb*EccC$_5$$^{DUF}$: *Mtb*PPE41 interfaces that might be compatible with the ESX-5 architecture. Interfaces of these models were thus analysed using jsPISA and PYMOL. Analysis of a first DUF:PPE41 interface revealed the prediction of three favourable contacts (two H-bonds and one salt bridge) and five unfavourable electrostatic interactions. Moreover, the interaction radar area estimated by jsPISA fitted within the 30% probability circle, which

supports a poor likelihood for the predicted interface (Krissinel, 2015). Analysis of the second DUF:PPE41 model revealed an interface exhibiting one favourable salt-bridge contact and an interaction radar area fitting within the 20% probability circle, which altogether indicated a low reliability for these modelled interfaces. Regarding the *Mtb*$EccC_5^{DUF}$:$EccD_5^{ULD}$ interaction, the control AF prediction carried out was able to model one of the two DUF:ULD interfaces observed in the *Mtb*ESX-5 structure, but the second *Mtb*$EccD_5^{ULD}$ molecule did not interact with DUF. Thus, and given these results, we did not pursue further predictions using more than two ULD monomers.

The AF predictions carried out using six copies of *Mtb*$EccC_5^{DUF}$ yielded an ensemble showing a ring structure and a *C*6 symmetry (see Table S1). However, analysis of this model revealed that DUF:DUF contacts took place through a region artificially generated by removal of the stalk and TM domains. Therefore, the contacts observed in this ensemble are unrealistic inter-monomer interactions. Summing up, the AF predicted models for the investigated interactions resulted in unreliable/unfeasible models. This could be due to a genuine absence of these interactions, but also to limitations in the modelling process that may arise from either the unreliability of the theoretical model itself or the requirement of other domains/components of the cytosolic complex that were not included.

## Table S1

| Interaction explored | Input $EccC_5$ sequence | Input partner sequence/s | $EccC_5$:partner stoichiometry | Comments |
|---|---|---|---|---|
| **Interactions modelled using a *Mtb*$EccC_5$ sequence corresponding to TM, stalk and DUF domains** | | | | |
| $EccC_5^{DUF}$:$EccD_5^{ULD}$ | | Residues 17-108 | 1:2 | Interactions modelled involved residues of both stalk and DUF domains of $EccC_5$ leading to ensembles non-compatible with ESX architecture |
| $EccC_5^{DUF}$:PE25:PPE41 | Residues 1-417 | Full-length | 1:1:1 | Interactions modelled involved residues of both stalk and DUF domains of $EccC_5$ leading to ensembles non-compatible with ESX architecture |
| $EccC_5^{DUF}$ hexamerisation | | – | 6 | Interactions modelled predicted an $EccC_5$ hexamerisation via the TM region exclusively, analogue to the observed in the membrane complex of *Mtb*ESX-5. |
| **Interactions modelled using a *Mtb*$EccC_5$ sequence corresponding to the DUF domain** | | | | |
| $EccC_5^{DUF}$:$EccD_5^{ULD}$ | | Residues 17-108 | 1:2 | Interactions modelled predicted one of the intra-protomer $EccC_5^{DUF}$:$EccD_5^{ULD}$ interactions, and no contacts between DUF and the second ULD. |
| $EccC_5^{DUF}$:PE25:PPE41 | Residues 123-417 (with residues 167-198 replaced by the sequence 'GSG') | Full-length | 1:1:1 | Two modelled complexes exhibited an ensemble compatible with the ESX-5 architecture but showing poor reliable DUF:effector interfaces due to poor contacts. |
| $EccC_5^{DUF}$ hexamerisation | | – | 6 | Hexameric models predicted consisted of four ensembles non-compatible with the ESX architecture and one ring-structure ensemble exhibiting a *C*6 symmetry axis |

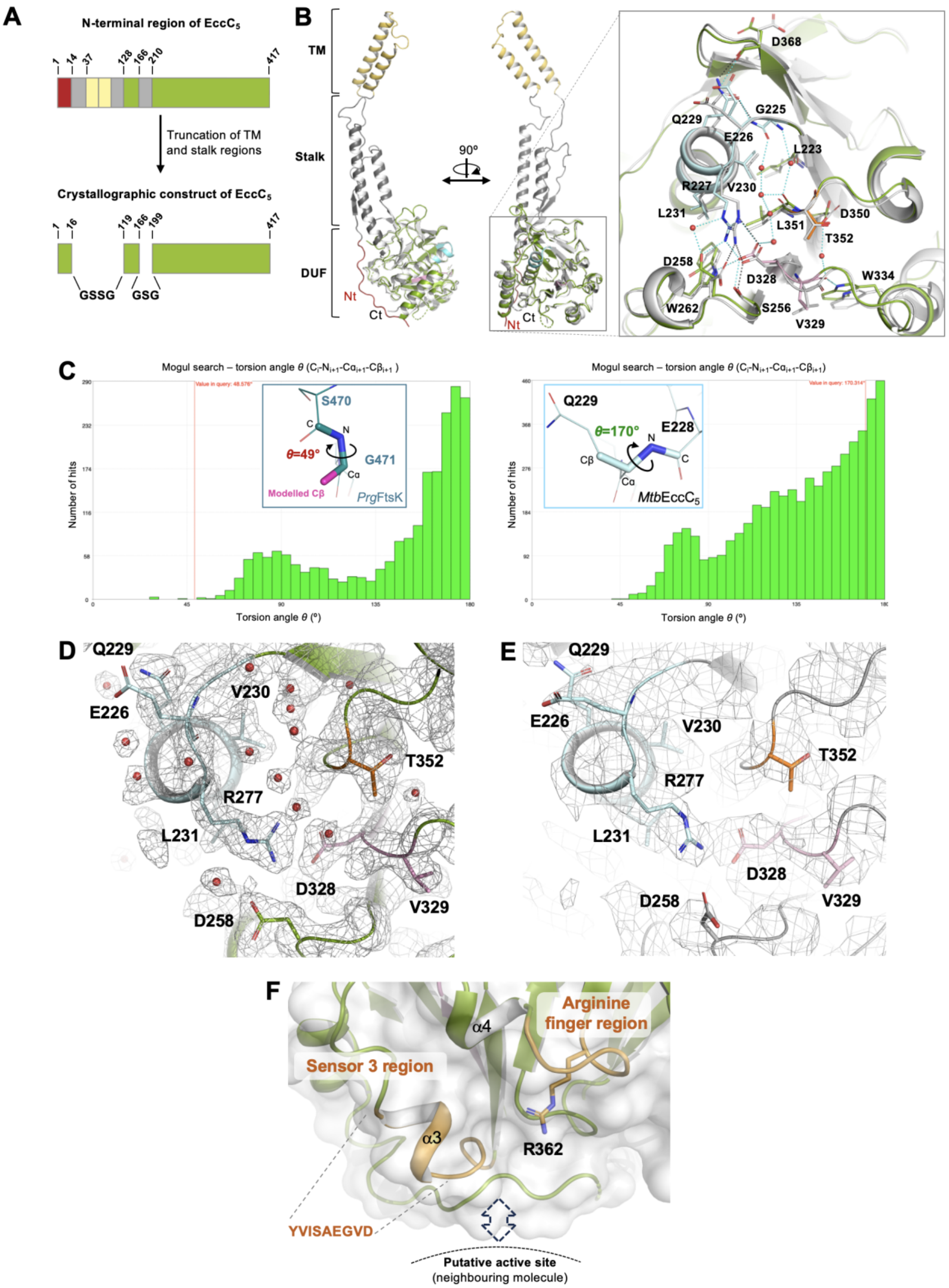
A
N-terminal region of EccC5
1 14 37 128 166 210 417
Truncation of TM and stalk regions
Crystallographic construct of EccC5
1 16 119 166 199 417
GSSG GSG
B
TM
Stalk
DUF
Nt
Ct
90º
D368
G225
Q229
E226
L223
R227
V230
L231
D350
L351
T352
D258
D328
W334
W262
S256
V329
C
Mogul search – torsion angle θ (Ci-Ni+1-Cαi+1-Cβi+1)
S470
θ=49°
G471
Modelled Cβ
PrgFtsK
Q229
θ=170°
E228
MtbEccC5
Number of hits
Torsion angle θ (º)
D
Q229
V230
E226
T352
R277
L231
D328
V329
D258
E
Q229
E226
V230
T352
R277
L231
D328
D258
V329
F
Arginine finger region
α4
Sensor 3 region
α3
R362
YVISAEGVD
Putative active site
(neighbouring molecule)

**Figure S1** (A) Scheme showing the architecture of the N-terminal region of *Mtb*EccC$_5$ formed by the TM (yellow), stalk (grey) and DUF (green) domains in the wild-type enzyme, and truncations introduced to remove TM and stalk domains in the crystallographic construct, where residues 17-118 and 167-198 are replaced by 'GSSG' and 'GSG' sequences respectively. (B) Structural superimposition of both crystallographic and cryo-EM *Mtb*EccC$_5^{DUF}$ models coloured as in Figure 2 and grey, respectively. In the case of *Mtb*EccC$_5^{CRYO}$, N-terminal, stalk and TM regions are coloured in red, dark grey and yellow, respectively. A zoomed view showing the structural conservation of both the Walker A and B motives in both models is shown (right), including the salt-bridge R227-D328 and the H-bond interaction E226-Q229 indicated as dashed lines coloured in blue (crystal model) and grey (cryo-EM model). (C) Mogul search results obtained for torsion angles $C_{S470}$-$N_{A471}$-$C\alpha_{A471}$-$C\beta_{A471}$ of *Prg*FtsK, when G471 is substituted by an alanine, and $C_{E228}$-$N_{Q229}$-$C\alpha_{Q229}$-$C\beta_{Q229}$ in *Mtb*EccC$_5^{DUF}$ crystal structure. (D) Detail of both Walker A and B motives in the *Mtb*EccC$_5^{DUF}$ crystal structure showing the 2Fo-Fc electron density map (contoured at a sigma value of 1.0). Colour code as in Figure 1. (E) Detail of both Walker A and B motives in *Mtb*EccC$_5^{DUF}$ model reported by cryo-EM showing the EM map at 3.27 Å resolution (PDB entry 7NPT) (contoured at a level value of 10.0). (F) Cartoon and surface representation *Mtb*EccC$_5^{DUF}$, showing details of the regions expected to allocate Arg-finger and Sensor 3 motives (highlighted in orange).

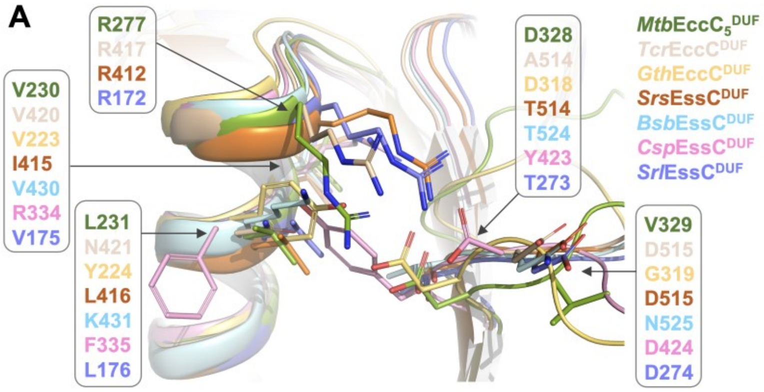


**Figure S2** (A) Structural superimposition showing a detail of the Walker A and B motives predicted for *Mtb*EccC$_5$$^{DUF}$, *Tcr*EccC$^{DUF}$, *Gth*EccC$^{DUF}$, *Srs*EssC$^{DUF}$, *Bsb*EssC$^{DUF}$, *Csp*EssC$^{DUF}$, and *Srl*EssC$^{DUF}$.

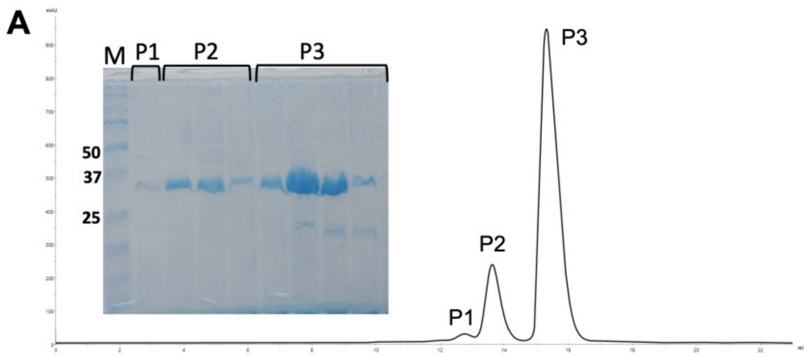


**Figure S3** (A) Size-exclusion chromatography profile obtained during the purification of the *Mtb*EccC$_5$$^{DUF}$ construct used in this study, and where hexameric (P1), trimeric (P2) and monomeric (P3) species are observed.
